# Application of Electric-Field-Optimized Augmented Reality-Guided Neuronavigation in Transcranial Magnetic Stimulation

**DOI:** 10.3390/jcm15072644

**Published:** 2026-03-31

**Authors:** Pia Ritter, Sascha Freigang, Antonio Valentin, Karla Zaar, Gernot Reishofer, Margit Jehna, Manuela Michenthaler, Sila Karakaya, Philipp Moser, Louis Frank, Robert Prückl, Stefan Schaffelhofer, Stefan Thumfart, Shane Matsune Fresnoza, Anja Ischebeck, Stefan Wolfsberger, Kariem Mahdy Ali

**Affiliations:** 1 Department of Radiology, Clinical Division of Neuroradiology, Vascular and Interventional Radiology, Medical University of Graz, 8036 Graz, Austria; 2 Department of Neurosurgery, Medical University of Graz, 8010 Graz, Austria; 3 Department of Basic and Clinical Neuroscience, Institute of Psychiatry, Psychology and Neuroscience (IoPPN), King’s College London, London SE5 8AF, UK; 4 Department of Radiology, Medical University of Graz, 8010 Graz, Austria; 5 Research Unit Medical Informatics, RISC Software GmbH, 4232 Hagenberg, Austria; 6 cortEXplore GmbH, 4020 Linz, Austria; 7 Department of Psychology, University of Graz, 8010 Graz, Austria

**Keywords:** augmented reality, language mapping, transcranial magnetic stimulation, electric-field simulation, object naming task, resting-state fMRI

## Abstract

**Background:** Navigated repetitive TMS (nrTMS) is widely used for non-invasive mapping of cortical functions. Methodological improvement might be achieved by optimizing coil positioning based on electric-field modeling and augmented reality (AR)-guided neuronavigation to enhance spatial targeting accuracy and stimulation-induced language errors. Therefore, we compared electric-field-optimized, AR-guided nrTMS with conventional nrTMS using manually planned coil positioning. **Methods:** Twenty-eight healthy subjects underwent two MRI-guided left hemispheric nrTMS language mapping sessions. Each session used 10 Hz stimulation at a 100% resting motor threshold applied for 1.5 s per region of interest (ROI) during a synchronized object naming task. ROIs were defined according to the Corina cortical parcellation system. Manually defined and electric-field-optimized coil placements obtained using SimNIBS (v4.1.0) were applied; the optimized session was assisted by AR goggles. The primary outcome was the quantitative and categorical differences in cortical regions mapped as language-eloquent. Resting-state fMRI was acquired to provide a reference for comparing nrTMS-derived language maps. **Outcomes:** Electric-field-optimized nrTMS did not result in an increase in positively mapped ROIs. A different distribution of language errors was observed between sessions. Manual mapping roughly followed the extracted resting-state language and motor networks, whereas electric-field-optimized mapping might correspond less. Optimized coil positions were not always practically feasible. AR guidance improved target location accuracy. **Conclusions:** While AR was a useful addition to the TMS experiment, electric-field optimization did not translate into significant behavioral differences. However, altered distribution of language errors can give insight into underlying neurophysiological processes of rTMS.

## 1. Introduction

Navigated repetitive Transcranial Magnetic Stimulation (nrTMS) is a method with broad applications in neuroscience and clinical research. As a non-invasive neuromodulation technique, it can be used for the mapping of cortical functions such as motor and language areas. Precise and time-controlled application of TMS stimuli is needed to achieve reliable and reproducible results. The biological response to the applied electrical field within the cerebral cortex remains poorly understood. TMS is considered a safe technique with few side effects [1,2].

In TMS studies, five key factors determine a successful trial: (1) definition of regions of interest (ROI) according to anatomical and functional properties; (2) characteristics of the induced electric field within the target region [3,4]; (3) the stimulation paradigm, e.g., high- or low frequency; (4) a suitable cognitive test and outcome assessment; (5) accurate and reproducible coil placement. Augmented reality (AR) is increasingly recognized for the use of medical devices such as neuronavigation [5,6]. Here we aimed to address two of these key factors outlined for TMS experiments (2) and (5). The latter might directly benefit from AR by improving availability of anatomical information to the examiner and aiding navigational accuracy, as well as decreasing examination time and therefore limiting priming effects induced by prior stimulation [7]. The former will be addressed by applying an electric-field simulation to inform coil position and orientation based on the modeled distribution of TMS-induced electric fields within each anatomical ROI. To date, electric-field modeling has been primarily validated in motor cortex applications, where individualized simulations that account for subject-specific anatomy show strong correlations with motor evoked potentials [8,9]. In this context, computational electric-field optimization has been shown to predict the optimal coil location and orientation to maximally stimulate a predefined cortical target, with predicted hotspot locations closely matching those identified through conventional manual mapping [10]. Beyond the motor cortex, electric-field optimization has been applied to therapeutic targets such as the prefrontal cortex; however, its transferability to language mapping has only been suggested, not empirically validated [9]; prospective validation in this domain remains lacking. To address this gap, the present study prospectively evaluates whether AR-guided, electric-field-optimized nrTMS improves language mapping performance compared to conventional manually planned nrTMS, with a particular focus on reproducibility and spatial distribution of mapping results in healthy subjects.

Specifically, we assessed whether electric-field-optimized coil placement, while targeting identical predefined cortical regions, results in differences in the frequency and distribution of language-related errors compared to conventional manual coil positioning during standard nrTMS language mapping using an object naming task (ONT) at 10 Hz stimulation frequencies [11]. It was hypothesized that increasing the effective electric field at the target would enhance neuromodulation effects and, in the context of language mapping, increase the likelihood of stimulation-induced language errors [4,12]. The corresponding null hypothesis was that electric-field-optimized coil placement would not lead to differences in the frequency or spatial distribution of stimulation-induced language errors compared with conventional manually planned coil positioning. To further contextualize these stimulation-induced effects, resting-state functional magnetic resonance imaging (fMRI) was acquired to provide an independent reference of large-scale language and motor networks. Previous work has shown that resting-state functional connectivity can explain how TMS effects propagate through the language network, with distinct connectivity profiles underlying different error types [13]. Integrating resting-state fMRI therefore allowed us to relate the spatial distribution of nrTMS-induced language errors to intrinsic functional connectivity patterns, and to assess differences in the correspondence with established networks between electric-field-optimized and manually planned coil positioning.

## 2. Materials and Methods

### 2.1. Participants

The trial was conducted as a prospective open study carried out at the Department of Neurosurgery, Medical University Graz. It was approved by the local ethics committee (ECS 115/2024) and was conducted in accordance with the ethical principles of the Declaration of Helsinki (as revised in Fortaleza, Brazil, 2013). Twenty-eight healthy adult volunteers (16 females, 12 males; mean age 28.39 ± 8.14 years) provided written informed consent prior to inclusion in the study. The inclusion criteria were having an age between 18 and 80 years, right-handedness, being a native speaker of German and having the ability to give informed consent. Contraindications were determined by MRI- and TMS safety standards. Subjects with epilepsy, chronic pain conditions or other neurological or psychiatric disorders were excluded. Participants were recruited between May and September 2025 via social media platforms and printed flyers posted on notice boards. An a priori power analysis conducted in G*Power 3.1.9.7 for a two-tailed paired-samples *t*-test, assuming a medium within-subject effect size (dz = 0.50), α = 0.05, and 80% power, indicated a required sample size of 34 participants. Due to recruitment and practical constraints during the study period, only 29 participants were initially recruited. Following the MRI session, one participant was excluded based on clinical exclusion criteria, resulting in a final sample of 28 participants.

### 2.2. Experimental Procedure

**Figure 1:**
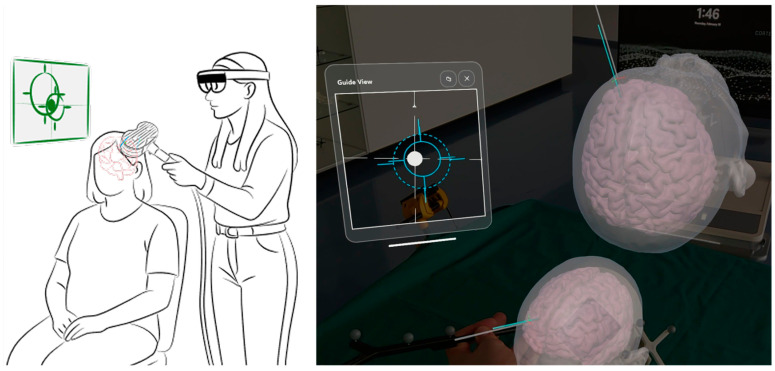
Augmented reality-guided visualization used during the electric-field-optimized nrTMS session. The experimenter viewed a three-dimensional model of the cerebral cortex projected into their visual field via augmented reality glasses (Microsoft HoloLens^®^). The current TMS coil position was displayed in real time together with a bull’s-eye visualization indicating the intended entry target.

All participants underwent a structural and functional MRI scan at the Department of Radiology. After MRI acquisition, each subject participated in two TMS sessions at the Department of Neurosurgery, separated by approximately one week (M = 10 days SD; 4.95). The first session is referred to as a conventional session, and the second as an optimized session. In the conventional session, neuronavigation was done using a Localite TMS Navigator (Localite GmbH, 53229, Bonn, Germany). The navigator, together with a Polaris Spectra infrared tracking camera (NDI, ON N2V 1C5, Waterloo, ON, Canada), was used to localize the coil with respect to the predefined ROIs. Additionally, in the conventional session, the CORTEXPLORER MED system (cortEXplore GmbH, 4320, Perg, Austria) simultaneously tracked and recorded the position of the TMS coil, based on the coil localization provided by the Localite Navigator, relative to the participant’s anatomy. In the optimized session, neuronavigation was performed using the CORTEXPLORER MED system. The system continuously tracked and updated the position of the TMS coil relative to the patient’s anatomy. The current coil position and target were displayed via AR-glasses (Microsoft HoloLens^®^, Redmond, WA, USA) worn by the experimenter during the optimized session. AR was employed by displaying a 3D model of the cerebral cortex freely in the visual field of the examiner. Live-tracking of the TMS coil and a bull’s-eye entry target visualization guided coil positioning in the electric-field-optimized session. See Figure 1 for a visualization of the AR guidance. Session order was fixed, with the conventional session always preceding the optimized session. Both sessions were performed by the same experimenter to avoid bias. In both sessions, TMS was performed using a MagPro X100 magnetic stimulator (MagVenture, 3520, Farum, Denmark) and a figure-of-eight coil (MCF B65). TMS-induced electric fields for optimizing coil positioning were modeled using SimNIBS v4.01 [14]. Based on patient-specific head models (generated from subject MR images) [15], SimNIBS (v4.1.0) uses state-of-the-art finite element methods to simulate the induced electric fields propagating through tissue types using realistic physical properties. An accurate coil model of the study TMS coil (MagVenture MCF B65) was readily available within SimNIBS (v4.1.0) [16]. Therefore, electric-field simulations were used to calculate the optimized TMS coil placement for each ROI [17], simulating the electric fields generated by thousands of possible coil placements and orientations around the cortical target. The optimization objective was to maximize the electric-field magnitude within each predefined ROI. Accordingly, we used SimNIBS (v4.1.0) with its default settings in combination with the auxiliary dipole method to efficiently evaluate approximately 1 million coil configurations and determine the optimal coil position and orientation [17].

### 2.3. TMS Protocol and Language Mapping

The TMS protocol and language mapping were identical for both the conventional and optimized sessions. During each nrTMS session, a standard 10 Hz paradigm applied for 1.5 s at a 100% resting motor threshold (RMT), determined at the abductor pollicis brevis muscle, was used. RMT was defined as the lowest intensity producing a motor evoked potential (MEP) in at least 50% of 10 single pulses [8]. ROIs were defined on the individual structural MRI in the left hemisphere according to the regions outlined by the Corina cortical parcellation system [18]. The coil was positioned tangentially to the scalp with its handle oriented approximately perpendicular to the target sulcus [19]. We planned to test each ROI three times with stimulation alternating systematically between frontal, parietal, temporal, and occipital regions. Due to limited coil accessibility, the three occipital regions and the most inferior temporal regions were excluded, resulting in 30 ROIs per participant being targeted for stimulation. During TMS application, a language task was performed consisting of 54 black-lined schematic pictures derived from the Boston Naming Test [20]. Prior to the first stimulation session, participants completed a baseline naming run to ensure that all stimuli were familiar and could be reliably named. All images were reviewed together with the participants and practiced until each item could be named fluently and without hesitation. Picture presentation and the triggering of the TMS stimulus employed the software “presentation^®^” (Version 23.0, Neurobehavioral Systems, Inc., Berkeley, CA, USA) on a common 15” laptop screen. Pictures were presented in randomized order within each session. The same set of pictures was used for the second session, again in randomized order. The interpicture interval (IPI) was response-contingent and manually triggered by the experimenter. After the participant provided a response, the next trial was initiated manually, resulting in slightly variable IPIs across trials. Picture presentation time (PPT) was 700 ms, and the picture-to-rTMS trigger interval (PTI) was 0 ms. Targets were interpreted as language-positive cortical areas when deficits were elicited and were subdivided into the following error categories: normal = A, hesitation = B, speech arrest = C, phonological paraphasia = D, semantic paraphasia = E, neologism = F, circumlocution = G, and dysarthria = H. Language errors were scored during the experiment by two observers in consensus. Each error was categorized and recorded using the corresponding predefined error category label. To reduce false-positive classifications, error categories were subdivided into major and minor deficits, allowing targets to be classified based on different thresholds of deficit occurrence. Major errors comprised speech arrest, semantic paraphasia, phonological paraphasia, and circumlocution. Minor errors were defined as hesitation and dysarthria. Major deficits were counted after a single occurrence, whereas minor deficits required occurrence in at least two out of three stimulations. This distinction was based on the assumption that minor errors, particularly hesitation, may have lower diagnostic specificity and may also arise from non-linguistic factors, such as TMS-related external facial muscle twitching.

### 2.4. Scanning Parameters and Preprocessing

Standard MRI sequences, commonly used in neuroscience studies, were acquired for neuronavigation and functional imaging. Measurements were carried out on a 3T MRI scanner (MAGNETOM Prisma fit, Siemens Healthineers, Erlangen, Germany) at the Department of Radiology, Medical University of Graz. The protocol included a T1-weighted MPRAGE sequence used as structural input for the navigated TMS sessions (TR = 1900 ms, TE = 2.22 ms, flip angle = 9°, voxel size = 1 × 1 × 1 mm^3^, matrix = 256 × 256 × 176, FOV = 256 × 256 × 176 mm^3^) and a T2-weighted turbo spin-echo (TSE) sequence (TR = 9320 ms, TE = 89 ms, flip angle = 140°, slice thickness = 3 mm, voxel size = 0.69 × 0.69 × 3 mm^3^, matrix size = 320 × 320 × 50, FOV ≈ 220 × 220 mm^2^). To identify relevant functional and structural language-related brain connectivity, resting-state BOLD images were obtained with a multiband echo-planar imaging sequence [21] (TR = 933 ms, TE = 33.4 ms, flip angle = 64°, matrix size = 96 × 96, FOV = 192 × 192 mm^2^, isotropic voxel size = 2 × 2 × 2 mm^3^; 96 axial slices, 338 volumes per run; total scan duration ≈ 5.3 min). Participants were positioned head-first and supine and were instructed to keep their eyes closed during the resting-state fMRI scan. Resting-state fMRI preprocessing was performed using FSL (FMRIB Software Library; v 6.0.7.13, Jenkinson et al., 2012 [22]) following the pipeline described by Pruim et al. [23]. All scans were visually inspected for artifacts prior to processing. Preprocessed time series were temporally concatenated across participants and entered into a group-level spatial independent component analysis (ICA) using FSL’s MELODIC, with the number of components fixed a priori at 30, a model order shown to reliably capture canonical large-scale networks while maintaining spatial stability [24,25,26]. Language network components were identified based on spatial overlap with core perisylvian regions (inferior frontal gyrus and posterior superior temporal gyrus) and left-hemisphere dominance, whereas the motor network was identified based on overlap with the precentral and adjacent sensorimotor cortex. Anatomical overlap was assessed using regions of interest from the Harvard–Oxford cortical atlas. For subsequent analyses, stimulation targets were classified as located inside or outside the language and motor networks based on voxel-wise overlap between a spherical region (radius = 5 mm; chosen to approximate the effective spatial resolution of TMS [27]) of interest centered on each target and the corresponding ICA components thresholded at Z = 2.3, a commonly used threshold for ICA-based network identification that balances sensitivity and specificity [28]. Targets showing any overlap were classified as inside the network. In total, 18 targets overlapped with the language network and seven with the motor network.

### 2.5. Outcomes and Explanation for Choice of Comparators

Primary outcome parameters included the comparison of positively and negatively mapped ROIs for conventional and electric-field-optimized coil positioning, as well as sub-analysis for respective language errors. Technical primary outcomes comprised the precision of coil positioning in each session and changes in electric-field strength at the cortical target between conventional and optimized sessions. Secondary outcomes included the comparison of error rates at stimulation targets located inside versus outside the language and speech–motor networks for each session.

### 2.6. Statistical Analysis

Statistical analyses were performed using paired *t*-tests when paired differences were normally distributed (Shapiro–Wilk test, *p* < 0.05) and Wilcoxon signed-rank tests otherwise. Differences in planned coil placement were quantified by computing Euclidean distances and root-mean-square errors (RMSE) between conventional and optimized scalp entry points for each target, as well as geodesic errors for orientational differences. Changes in induced electric-field magnitude at the target were expressed as percentage increases relative to conventional placement. In addition, positional coil accuracy during the TMS session, defined as the deviation between the actual and the planned coil placement at the entry point, was compared between conventional and optimized sessions and assessed for statistical significance using the Mann–Whitney U test due to unequal numbers of successfully stimulated targets across sessions. All measures were reported as the median and interquartile range (IQR) due to non-normal data distribution. To assess whether variability in the electric field increases influenced TMS outcomes, we performed an analysis using a generalized linear mixed model (GLMM) with a binomial logit link. The model predicted positive stimulation in the optimized session. Fixed effects included the percentage increase in electric-field magnitude and positive stimulation in the conventional session, while random intercepts for subjects and regions accounted for repeated measurements and inter-individual variability. Including the percentage increase as a continuous predictor allowed us to explicitly test whether heterogeneity in the electric field increased across regions and participants moderated the likelihood of positive stimulation.

Additionally, Wilcoxon signed-rank tests were used to compare the absolute number of successfully delivered stimulations between the conventional and optimized sessions, as well as to compare the ratio of minor versus major language errors between sessions. Paired *t*-tests were used to compare overall error rates between sessions and to assess differences in error rates for stimulation targets located inside versus outside the identified language and motor network components. Statistical significance was defined as *p* < 0.05.

## 3. Results

Of the 28 participants, 27 completed both sessions, while one completed only the conventional session. The reason for the omitted session was unrelated to the experiment. Initially, we aimed to reproduce the stimulation sequence of the conventional session during the optimized session. However, the number of stimulated targets was significantly lower in the optimized session than in the conventional session (*Z* = −2.36, *p* = 0.018, *r* = 0.45), corresponding to 630 and 655 stimulations, respectively.

The optimization procedure resulted in noticeable differences in coil positioning between sessions. Planned coil positions differed statistically significantly (*p* < 0.05), with a median Euclidean distance of 4.62 mm (IQR = 4.14 mm) and median RMSE of 2.67 mm (IQR = 2.38 mm) between entry points in the conventional and optimized sessions. Also, a median orientational difference of 36° (IQR = 64.20°) was obtained. Positive stimulation in the conventional session strongly predicted positive stimulation in the optimized session (β = 1.77, OR = 5.88, 95% CI = 2.37–14.55, *p* < 0.001), corresponding to roughly a sixfold increase in the odds of observing positive stimulation. The percentage increase in the electric field showed substantial heterogeneity across regions and participants (median = 6.7%, IQR = 11.54%) but did not significantly influence positive stimulation in the GLMM (β = −0.048, OR = 0.95, 95% CI = 0.91–1.00, *p* = 0.050), and no trend was observed of larger increases yielding more language errors. Random effects indicated substantial variability across subjects and moderate variability across regions.

**Figure 2:**
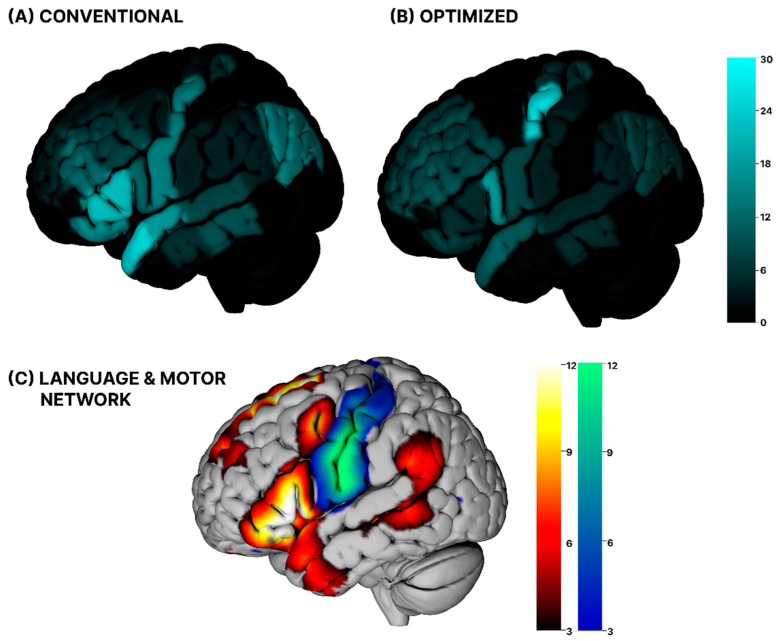
Error heatmaps of (**A**) the conventional session and (**B**) the optimized session showing the spatial distribution of stimulation-induced language errors across cortical regions. For each stimulation target, color intensity represents the local error rate (% of stimulations eliciting an error), with brighter colors indicating higher error rates. (**C**) Resting-state language and motor networks extracted by group independent component analysis (ICA). The language network is shown in red–yellow, involving left perisylvian frontal and temporal regions, while the motor network is shown in blue–green, covering the precentral and postcentral areas. Maps are displayed as Z-scores thresholded at Z ≥ 3, with values up to Z = 12 indicating the strongest component weights.

Descriptively, the conventional session showed a higher overall proportion of positive stimulation effects than the optimized session (7.3% vs. 6.6%); however, this difference did not reach significance (*t*(26) = 0.65, *p* = 0.52). With respect to the types of positive stimulation effects, both sessions showed a mixture of major and minor errors across cortical regions. No significant differences were found regarding the subclassification of language errors (*Z* = −0.13, *p* = 0.895, in both sessions 55% major and 45% minor deficits). Regarding cortical distribution, positive stimulations occurred most frequently in temporal and frontal regions under conventional stimulation. Optimized stimulation resulted in redistribution of positive areas towards central regions, while frontal and temporal regions showed lower rates compared with the conventional session (see Table 1 and Figure 2). Central regions were the only cortical area in which the optimized sessions showed a descriptively higher rate of positive stimulations compared with the conventional session. Analyses by cortical region and error type are reported descriptively only because of low event counts. Regional error rates for both sessions are reported in Table 1.

Visual comparison of the spatial distribution of stimulation-induced errors with resting-state fMRI-derived language and motor networks appeared to show higher alignment with the canonical network patterns in the conventional session than in the optimized session (see Figure 2). Across sessions, error rates were descriptively higher for targets located within functional language and motor networks compared with targets outside these networks (7.7% vs. 5.4%), although this difference did not reach statistical significance (*t*(26) = 1.54, *p* = 0.14; inside: *M* = 0.08, *SD* = 0.08; outside: *M* = 0.05, *SD* = 0.08). Session-specific comparisons similarly revealed no statistically significant differences. In the conventional session, error rates within functional networks were 7.7% compared with 6.6% outside the networks (*t*(26) = 0.53, *p* = 0.60; inside: *M* = 0.08, *SD* = 0.08; outside: *M* = 0.07, *SD* = 0.09). In the optimized session, error rates within functional networks were 7.5% compared with 4.3% outside the networks (*t*(26) = 1.70, *p* = 0.10; inside: *M* = 0.08, *SD* = 0.10; outside: *M* = 0.04, *SD* = 0.08).

**Figure 3:**
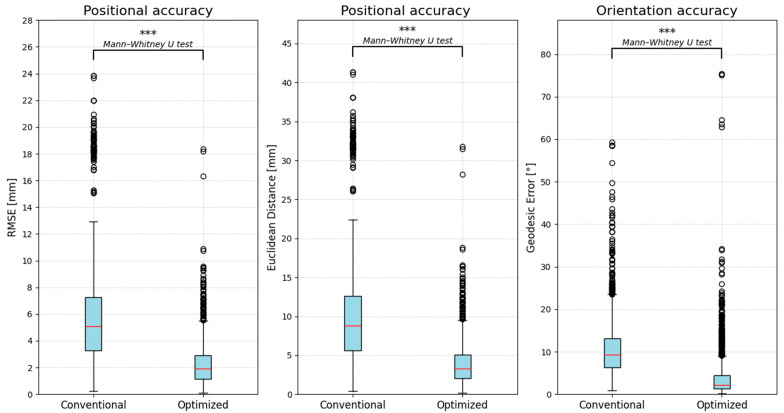
Comparison of coil positioning accuracy between the conventional and optimized, augmented reality-guided sessions. Positional error at the coil entry point (RMSE and Euclidean error) and orientation error quantified by the geodesic coil angle are shown. Significantly reduced positional and angular errors were obtained with AR-guided navigation compared with the conventional session. Asterisks denote statistical significance: *** *p* < 0.001.

Regarding the use of the Microsoft Hololens^®^ to facilitate AR-guided neuronavigation, both examiners reported overall positive feedback. Specifically, availability of data in the visual field and easier target localization facilitated by the bull’s-eye visualization were mentioned. Compared with the conventional session, AR-guided navigation was associated with statistically significantly (*p* < 0.05) improved coil positioning accuracy (see Figure 3). Positional error at the entry point, quantified by RMSE (median 1.9 mm vs. 5.1 mm; IQR 1.7 mm vs. 4.0 mm), Euclidean error (median 3.3 mm vs. 8.8 mm; IQR 3.0 mm vs. 6.9 mm), and orientation accuracy measured as the geodesic error (median 2.2° vs. 9.3°; IQR 3.1° vs. 6.9°), were all significantly lower in the AR-guided sessions.

## 4. Discussion

The present study compared electric-field-optimized with conventional nrTMS language mapping with a primary focus on changes in error rate and the spatial distribution of stimulation-induced language errors. Additionally, we evaluated the feasibility and utility of augmented reality (AR)-assisted neuronavigation to guide the electric-field-optimized stimulation session. It was hypothesized that higher effective electric fields at predefined cortical targets would be associated with increased error rates. In TMS research, the magnitude of the induced electric field at the cortical target is widely regarded as an important determinant of neuronal activation or inhibition and is therefore frequently used as an optimization criterion in computational modeling studies [29,30]. However, electric-field magnitude represents only one of several factors influencing stimulation efficacy, alongside parameters such as field orientation relative to cortical geometry [3]. Stimulation intensity has been shown to correlate with induced motor-sensory and behavioral effects, although this relationship seems to be heterogenous [31,32,33].

By design, the optimization algorithm increased the modeled electric field at the cortical target, which inherently required substantial changes in coil positioning, including a displacement of the coil entry point by more than 4 mm and pronounced differences in coil orientation. In contrast to our assumption that increased field strength would lead to more stimulation-induced language deficits [19,34], no significant increase in language errors was observed between the conventional stimulation session and the optimized session. However, when considering the spatial distribution of stimulation-induced errors, divergent patterns emerged across sessions. In the conventional session, errors showed greater visual correspondence with the canonical resting-state language and motor network based on qualitative visual inspection. A focal increase in error rates in the conventional session was observed primarily in the inferior frontal gyrus, anterior/superior temporal regions, and the precentral cortex. This pattern is consistent with prior TMS language mapping studies regarding the perisylvian and motor regions as error-prone stimulation sites, which are part of the established language and motor network [35,36,37]. A minor elevation of error rates, albeit less pronounced, was also observed in the angular gyrus. Despite its well-established involvement in language-related cognition, we did not identify the angular gyrus as part of the language network component. This is in-line with previous studies showing its characterization as a heteromodal connector hub associated with other large-scale networks such as the default mode network [38,39,40,41]. In rTMS language mapping studies, stimulation of the angular gyrus has been reported, but is not among the most consistently identified error-prone sites relative to classical perisylvian regions [19,36,42].

In the optimized session, based on qualitative visual inspection, the spatial distribution of stimulation-induced errors showed a weaker visual correspondence with canonical resting-state language and motor network patterns compared with the conventional session. In a descriptive comparison of the observed error rates, the lower overall error rate under optimized stimulation appears to be driven primarily by fewer errors at targets outside functional networks, whereas error rates within language and motor networks were similar between sessions (inside: 7.7% vs. 7.5%; outside: 6.6% vs. 4.3%). While this pattern could tentatively be interpreted as a reduction in unspecific or off-network effects, the spatial distribution of errors under optimized stimulation argues against more precise functional targeting, as it showed substantially less correspondence with regions traditionally considered critical for language function or with the ICA-derived language and motor networks. Additionally, Gomez-Tames et al. [43] have demonstrated that the modeled maximal electric-field strength is consistently higher in central regions such as the M1 area compared to other cortical areas, which may direct electric-field optimization toward these areas and could explain the shift towards these regions in the optimized session. However, we note that this would not explain task-specific findings in regard to language deficits. More generally, we note that comparisons with the rs-fMRI-derived networks in the present analysis were based only on qualitative visual inspection. Quantitative approaches, such as spatial permutation tests or coordinate-based analyses, would provide a more rigorous statistical evaluation of target–network overlap [44,45].

Despite substantial heterogeneity in electric field increases across regions and participants, we did not observe a relationship between larger field increases and a higher likelihood of language errors. This finding suggests that electric-field magnitude alone may not be the primary determinant of the behavioral effects of TMS. Instead, language disruption may depend more strongly on factors such as the intrinsic functional relevance of the stimulated region, its baseline responsiveness to TMS, as well as coil orientation and the underlying cortical geometry. While electric-field magnitude at the target location contributes to neuronal activation [4,46], other models suggest that stimulation efficacy depends critically on the orientation and spatial distribution of the induced electric field relative to cortical anatomy, rather than on field magnitude alone [38,39,40]. For example, building on the cortical column cosine model (C3), which emphasizes electric-field components perpendicular to the cortical surface as particularly relevant for neuronal activation, Janssen et al. [47] demonstrated that maximizing total electric-field magnitude does not necessarily maximize this perpendicular field component. Consequently, following this framework and our present findings, optimization strategies based solely on overall field strength may not translate into corresponding functional effects [47]. Moreover, another factor to consider is that the increase in electric-field magnitude achieved in the optimized condition may have been insufficient to produce measurable additional behavioral effects. Thus, the absence of significantly greater language disruption does not necessarily indicate that electric-field magnitude is irrelevant for stimulation efficacy, but may reflect that the achieved increase remained below a threshold required for additional modulation. That said, behavioral effects were descriptively lower rather than higher in the optimized condition, which argues against a simple dose–response relationship and instead supports the interpretation that factors such as coil positioning or field orientation may be more critical determinants of TMS efficacy.

From a functional perspective, early interleaved TMS–fMRI studies demonstrated that increasing stimulation intensity led to progressively stronger BOLD responses not only at the cortical target but also in regions remote from the stimulation site, indicating that higher intensities engage distributed neural responses beyond the targeted cortex [31,48,49]. Although relationships between stimulation intensity and activation in more distant brain regions have been reported only sparsely in more recent TMS research, Riddle et al. [50] demonstrated intensity-dependent modulation of BOLD responses in anatomically connected distal targets, particularly within fronto-striatal circuits. In several TMS studies, increased engagement of distributed network components has been interpreted as reflecting the recruitment of parallel or compensatory processes [51,52,53,54,55]. For example, Hartwigsen et al. [53] demonstrated that focal perturbation of the angular gyrus induced rapid upregulation of functionally related regions, with the extent of network reorganization being directly associated with preserved behavioral performance. Thus, higher stimulation intensities may have engaged additional neural processes that could have allowed compensatory responses, thereby attenuating or obscuring measurable behavioral effects despite pronounced neural modulation. Importantly, relatively few studies have systematically examined the relationship between stimulation intensity and the spatial focality of TMS effects, and even fewer have directly linked intensity-dependent changes in network engagement to behavioral outcomes. Consequently, interpretations regarding intensity-related shifts from local to more distributed or compensatory network effects should be considered tentative and require direct empirical testing in future studies [56].

Beyond physiological and modeling-related considerations, practical limitations of the optimized protocol should be acknowledged. For some targets, optimization led to coil orientations with reduced practical feasibility. In particular, maintaining stable positioning of the TMS coil was more challenging and time-consuming in upward-oriented handle configurations than in standard, typically anterior–posterior oriented setups. Furthermore, altered coil positioning in the optimized session significantly reduced the number of successfully stimulated targets relative to the conventional session, partly due to anatomical interference between the coil handle and the shoulder (downward-facing coil handle) and reduced visibility of coil-mounted tracking markers; this primarily affected posterior inferior temporal regions. While participant discomfort also contributed to the overall number of excluded targets, it cannot be attributed to the optimized setup, as targets excluded due to discomfort in the conventional session were not stimulated in the optimized session. Consequently, the present data does not allow conclusions as to whether electric-field optimization was associated with increased stimulation-related discomfort. As a possible approach for future work, optimization scripts could be extended to include geometric feasibility checks, explicitly considering whether coil positions are practically feasible given the subject-specific anatomy and possible hardware constraints. Such an approach would trade a slight reduction in the maximum electric-field strength at the cortical target location for improved practical feasibility and stability of coil positioning during optimization. Thereby, the number of targets that can be stimulated could be increased while still maintaining sufficient physiological efficacy.

Regarding the use of AR, operators reported overall positive feedback, particularly with respect to the utility of the three-dimensional cortical visualization and the bull’s-eye guidance. Moreover, the results demonstrated that AR-guided neuronavigation significantly improved target localization accuracy. A limitation was observed for the bull’s-eye visualization in combination with upward-facing handle orientations, where the direction mapping resulted in inverted feedback. Although this was perceived as counter-intuitive by operators, it reflects a technical implementation issue that could be readily addressed in future versions or eliminated altogether by avoiding upward-facing handle orientations. While the current implementation of the AR system already provides meaningful benefits, future developments offer additional opportunities to further enhance its functionality. For instance, projecting the three-dimensional brain model directly onto the participant’s head surface may further facilitate target localization and reduce examiner workload, particularly for complex or nonstandard coil orientations.

In summary, the present findings indicate that electric-field optimization based solely on field magnitude may be insufficient to enhance stimulation outcomes. Nevertheless, automated and model-based planning approaches remain attractive for clinical TMS applications, as they increase objectivity, reduce operator dependency, and improve time efficiency by eliminating the need for manual planning. Future work may therefore benefit from refining the optimization target itself. Rather than maximizing overall electric-field strength, optimization criteria could incorporate field components oriented parallel or perpendicular to deeper neuronal pathways, for example, informed by subject-specific tractography. Although the present results did not demonstrate improved outcomes with the applied optimization strategy, electric-field-based optimization remains a promising avenue for clinical applicability once physiologically more appropriate optimization targets are identified. The integration of AR-based neuronavigation proved beneficial, both in terms of examiner-reported subjective experience and in improving target localization accuracy. From a practical perspective, the implementation of these technologies also requires consideration of economic factors; while exact costs depend on the specific hardware configuration and licensing model, XR-based tracking and neuronavigation systems are typically available in the mid-five-figure to low six-figure price range. In our experience, however, the system proved to be highly intuitive, requiring minimal training and no substantial additional preparation time, which may facilitate integration into existing research and clinical workflows and help offset initial costs over time.

Several limitations of the study need to be acknowledged. The study included 28 participants compared to the a priori target of 34 participants based on power calculations. While the use of generalized linear mixed-effects models incorporating multiple observations per participant likely improved statistical efficiency, the study may have been underpowered to detect small-to-medium effects in some analyses. Accordingly, non-significant findings should be interpreted with appropriate caution. A further limitation of the present study is that we did not explicitly examine whether inter-individual differences in cortical morphology (e.g., sulcal depth, cortical curvature, or gyral orientation) influence TMS-induced electric-field distributions or stimulation outcomes. Addressing this question would require dedicated morphometric analyses in larger cohorts to ensure sufficient statistical power. Some additional aspects of the study design should be considered. First, while the baseline object naming task ensured stimulus familiarity prior to the first session, the fixed within-subject order of stimulation conditions does not allow potential order effects, such as increasing familiarity with the task procedure or TMS sensation across sessions, to be fully excluded. Second, the optimized session involved several simultaneous methodological modifications, including electric-field-optimized coil positioning and orientation, the use of a different neuronavigation system, and AR-based visual guidance, such that the present study design does not allow the individual contribution of these components to be disentangled in all respects. Third, language lateralization was inferred from handedness rather than directly assessed. Future studies could validate hemispheric dominance using a lateralization index derived from functional data.

Regarding the generalizability of the present study, it should be noted that the experiments were conducted in young and healthy volunteers. Consequently, the applicability of these findings to clinical populations is limited. However, studying healthy participants allows for a controlled experimental setting without disease-related variability or anatomical distortion. Whether the optimization procedure may yield different results in clinical populations, for example in the presence of altered cortical anatomy, shifted activation thresholds, or functional reorganization, remains to be investigated in future patient studies.

## 5. Conclusions

We conclude that augmented reality (AR) guidance enhances the practical implementation of navigated nrTMS language mapping by improving coil placement accuracy and facilitating operator workflow. We further acknowledge the clinical advantages offered by electric-field-based optimization, particularly with respect to increased objectivity and reduced planning time. However, the present results suggest that optimization strategies based solely on absolute electric-field strength may be insufficient, underscoring the need for more physiologically informed optimization targets. Maximization of the induced electric field did not increase the overall percentage of positively stimulated targets, but rather, was associated with a distinct shift in the spatial distribution of stimulation-induced language errors, showing a visually weaker correspondence with resting-state fMRI-derived language and motor networks compared with conventional, manually planned TMS. While the combined use of electric-field-based planning and AR guidance still requires further refinement, it represents a promising avenue for future TMS research and clinical applications.

## Figures and Tables

**Table 1:** Cortical distribution error rates and distribution of major and minor stimulation-induced deficits for the conventional and optimized nrTMS sessions.

% Minor Deficit	% Major Deficit	% Error Rate	Region
Conventional Session			
57.90%	42.10%	7.9% (19/239)	Frontal
54.50%	45.50%	5.4% (6/112)	Parietal
37.50%	62.50%	9.8% (16/164)	Temporal
58.30%	41.70%	7.1% (12/168)	Central
45.30%	54.70%	7.8% (53/683)	Total
Optimized Session			
42.90%	57.10%	6.5% (14/216)	Frontal
0.00%	100%	2.8% (3/108)	Parietal
42.90%	57.10%	4.9% (7/144)	Temporal
56.30%	43.80%	9.8% (16/162)	Central
45.00%	55.00%	6.3% (40/630)	Total

## Data Availability

The data supporting the findings of this study are not publicly available due to privacy, ethical, and legal restrictions governing the collection and use of human participant data. The applicable regulatory framework does not permit public deposition of the dataset. No new publicly archived datasets were generated in this study.

## Abbreviations

The following abbreviations are used in this manuscript:

3T	3 Tesla
AR	Augmented Reality
BOLD	Blood-Oxygen-Level-Dependent (signal)
ECS	Ethics Committee reference (Ethics Committee Study/Submission number)
FMRIB	Functional Magnetic Resonance Imaging of the Brain
FOV	Field of View
FSL	FMRIB Software Library
fMRI	Functional Magnetic Resonance Imaging
GE-EPI	Gradient-Echo Echo-Planar Imaging
ICA	Independent Component Analysis
IFG	Inferior Frontal Gyrus
IPI	Interpicture Interval
IQR	Interquartile Range
M	Mean
M1	Primary Motor Cortex
MELODIC	Multivariate Exploratory Linear Optimized Decomposition into Independent Components
MEP	Motor Evoked Potential
MPRAGE	Magnetization-Prepared Rapid Acquisition Gradient Echo
MRI	Magnetic Resonance Imaging
NBS	Neurobehavioral Systems
nrTMS	Navigated Repetitive Transcranial Magnetic Stimulation
ONT	Object Naming Task
*p*	*p*-value
PPT	Picture Presentation Time
PTI	Picture-to-rTMS Trigger Interval
rTMS	Repetitive Transcranial Magnetic Stimulation
RMSE	Root-Mean-Square Error
RMT	Resting Motor Threshold
ROI	Region of Interest
SD	Standard Deviation
SimNIBS	Simulation of Non-Invasive Brain Stimulation (software) v4.1.0
TE	Echo Time
TMS	Transcranial Magnetic Stimulation
TR	Repetition Time
TSE	Turbo Spin Echo
U	Mann–Whitney U statistic
Z	Z-score
Z (Wilcoxon)	Z-statistic (Wilcoxon signed-rank test)
